# WISP2 exhibits its potential antitumor activity via targeting ERK and E-cadherin pathways in esophageal cancer cells

**DOI:** 10.1186/s13046-019-1108-0

**Published:** 2019-02-26

**Authors:** Da-Min Chai, Yan-Zi Qin, Shi-Wu Wu, Li Ma, Yuan-Yuan Tan, Xiang Yong, Xiao-Li Wang, Z. Peter Wang, Yi-Sheng Tao

**Affiliations:** 1 Department of Pathology, the First Affiliated Hospital of Bengbu Medical University, Bengbu Medical College, Changhuai road 287#, Bengbu, Anhui, 233000 People’s Republic of China; 2 Department of Biochemistry and Molecular Biology, School of Laboratory Medicine, Bengbu Medical College, Anhui, 233030 China; 3 Department of Pathology, Beth Israel Deaconess Medical Center, Harvard Medical School, 330 Brookline Ave, Boston, MA 02215 USA

**Keywords:** Esophageal cancer, WISP2, Proliferation, Invasion, Migration

## Abstract

**Backgrounds:**

Emerging evidence has demonstrated that WISP2 is critically involved in cell proliferation, migration, invasion and metastasis in cancers. However, the function of WISP2 in esophageal squamous cell carcinoma (ESCC) is largely unclear. Therefore, we aim to explore the effects and the potential mechanism of WISP2 on proliferation and motility and invasion of ESCC cells.

**Methods:**

Cell proliferation was detected by MTT assay and apoptosis was measured by FACS in ESCC cells after WISP2 downregulation and overexpression. Cell migration and invasion were analyzed by wound healing assay and transwell migration assay, respectively. The expression of ERK-1/2, Slug and E-cadherin was measured by Western blot respectively. IHC was performed to measure the expression of WISP2 in ESCC tissues.

**Results:**

WISP2 overexpression is associated with survival in ESCC patients. WISP2 overexpression inhibited cell growth and induced cell apoptosis, suppressed cell migration and invasion in ESCC cells. Moreover, WISP overexpression retarded tumor growth in mouse model. WISP2 downregulation enhanced cell growth, inhibited apoptosis, promoted cell migration and invasion in ESCC cells. Mechanistically, WISP2 exerts its tumor suppressive functions via regulation of ERK1/2, Slug, and E-cadherin in ESCC cells.

**Conclusions:**

Our findings suggest that activation of WISP2 could be a useful therapeutic strategy for the treatment of ESCC.

## Background

Esophageal cancer is the common malignant neoplasm, which arises from the mucosa or gland of the esophagus. An estimated 17,290 patients will be diagnosed with esophageal cancer, and 15,850 people will die due to this disease in 2018 in the United States [1]. The reasons of esophageal cancer development are unclear. It has been reported that dietary habits, environmental factors, stress, and genetic mutations could contribute to esophageal cancer [2]. The current treatments of esophageal cancer include surgical resection, chemotherapy, radiotherapy, or combined strategy [3, 4]. Due to the metastatic incidence at diagnosis, drug resistance after chemotherapy and recurrence after surgery, long-term survival is still low in patients with esophageal cancer [5]. Early detection and treatment of esophageal cancer is a promising strategy to improve the survival rate in esophageal cancer patients. However, esophageal cancer patients often exhibit the onset of symptoms at diagnosis, which leads to poor prognosis. Alternatively, identification of molecular mechanism underlying esophageal tumorigenesis could be helpful for the better treatment of esophageal cancer patients.

WISP2 (WNT1 inducible signaling pathway protein 2) was reported to be upregulated in the mammary epithelial cells transformed by the Wnt-1 oncogene [6]. Subsequently, WISP2 was found to be directly regulated by the estrogen receptor in human breast cancer cells [7]. One study further identified that WISP2 expression was enhanced by serum and correlated with serum-induced cell proliferation in breast cancer cells, demonstrating that WISP2 could enhance cell proliferation in breast cancer [8]. Moreover, WISP2 waas reported to have higher expression in breast cancer patients at late stages with metastasis, indicating that WISP2 plays an oncogenic role in breast cancer [9]. Controversially, several studies revealed that WISP2 is a tumor suppressor in breast cancer [10, 11]. For example, one group observed that WISP2 level is inversely correlated with lymph node positivity. Notably, WISP2 was a negative regulator of migration and invasion via regulation of MMP-2 (matrix metalloproteinase-2), and MMP-9 in breast cancer cells [10]. Consistently, WISP2 overexpression led to cell cycle arrest at G1/G1 phase, inhibited cell growth, suppressed tumor growth in the xenograft model in breast cancer [11]. Additionally, WISP2 downregulation triggered EMT in breast cancer cells, while WISP2 overexpression suppressed cell proliferative and invasive phenotypes in breast cancer cells [12]. In gastric cancer patients, WISP2 expression is associated with tumor stage, differentiation status, and overall survival [13]. Another group reported that WISP2 was highly expressed in gastric tissues compared to adjacent normal tissues [14]. It has been reported that lower WISP2 expression was observed in pancreatic cancer tissues [15]. These studies indicated that WISP2 plays an important role in the development and progression of human cancers.

Emerging evidence has shown that WISP2 is critically involved in tumorigenesis in various types of human cancers. However, there are no available reports to show the function of WISP2 in ESCC. Therefore, in the current study, we explored the functions of WISP2 in ESCC cells via upregulation and downregulation of WISP2, including cell growth, apoptosis, migration and invasion. Moreover, we determined mechanisms of WISP2-invloved in ESCC progression. Our study will provide the direct evidence for role of WISP2 in ESCC.

## Materials and methods

### Human ESCC samples

The 216 cases of paraffin-embedded ESCC, 60 cases of normal esophageal epithelium, and 20 cases of esophagitis were collected at the time of surgery between 2006 and 2008 at the first affiliated hospital to Bengbu Medical College (Anhui, China). The clinical information of patients is described in detail in Table 1. ESCC patients were treated with surgery, but not with chemotherapy or radiation therapy. The 28 cases of ESCC specimens were frozen in liquid nitrogen immediately and stored at − 80 °C. The study was approved by the Ethics Committee of Bengbu Medical College.

**Table 1:** Expression of WISP2 in ESCC and the relationship with clinicopathological parameters.

Parameter	cases	WISP2			χ^2^	*P*
		+	–	Positive(%)		
Gender						
Male	121	44	77	36.36		
Female	95	33	62	34.74	0.06	0.89
Age(years)						
≤ 60	86	35	51	40.70		
>60	130	42	88	32.31	0.21	0.25
Tumor grade						
I	50	20	30	40.00		
II	78	29	49	37.18		
III	88	28	60	31.82	1.78	0.62
Infiltration depth						
Under serous layer	99	71	28	71.72		
Serous layer	117	6	111	0.51	103.65	< 0.01
Lymph node metastasis						
Yes	112	2	110	1.79		
No	104	75	29	72.12	116.27	< 0.01
TNM stages						
I,II stages	88	71	17	80.68		
III,IV stages	128	6	122	4.69	131.28	< 0.01
Diameter(cm)						
<4	97	36	61	37.11		
≥ 4	119	41	78	34.45	0.69	0.78
General type						
Ulcerative type	67	21	46	23.8		
Medullary type	97	34	63	23.8		
Mushroom type	41	18	23	18.2		
Constrictive type	11	4	7	25.0	0.33	0.96
Location						
Top	28	14	14	50.00		
Middle	116	34	82	29.31		
Bottom	72	29	43	40.28	5.22	0.07

**Table 2:** Expression of WISP2 in normal esophageal mucosa and ESCC

Parameter	Total	WISP2 expression			χ^2^	*P* value
		+	–	Positive (%)		
Normal mucosa	60	31	29	51.66	5.78	0.02
Tumor tissue	216	77	139	35.65		

### Histological sections and immunohistochemistry

Immunohistochemical studies were performed to determine the expression of WISP2 in tumors as described before [16].

### Cell culture

Human ESCC cell lines, EC9706 and Eca109 cells, were purchased from the Cell Resource Center, Shanghai Institutes for Biological Sciences (Shanghai, China) and cultured in Dulbecco’s modified Eagle’s medium (DMEM) containing 10% fetal bovine serum (FBS, Sigma, USA)**.** Subcultures were prepared using 0.05% trypsin solution (Invitrogen, Carlsbad) and seeded in 6- or 96-well tissue culture plates.

### Transfection

The transfection of WISP2 plasmid or WISP2 siRNA or control siRNA was conducted using Lipofectamine 2000 reagent (Invitrogen, Carlsbad, CA, USA). All WISP2 siRNAs were purchased from shanghai GenePharma in China.

### Real-time quantitative PCR

The total RNA of ESCC transfected cells in each group was isolated with Trizol (Invitrogen, Carlsbad) and reverse-transcribed to cDNA with Reverse Transcription System [17]. Quantitative real-time PCR was performed on an ABI 7900 System with SYBR green (Takara, China). Primers of WISP2 were as follows: TGC TGC CCT GAG TGG GTG (forward, 5′-3′) and GAA GCG GTT CTG GTT GGA C (reverse, 5′-3′). Transcripts were quantified with GAPDH (glyceraldehyde 3-phosphate dehydrogenase) as an internal standard.

### Cell viability assay

The ESCC cells were seeded in 6-well plates overnight and transfected with WISP2 plasmid or its siRNA, for 48 h. Then, ESCC cells were trypsinizated and seeded in 96 well plates for 72 h**.** Cell viability was detected by MTT assay as described previously [18].

### Apoptotic cells measurement

Apoptotic cell death was detected using the annexin V-FITC / propidium iodide (PI) staining kit (BestBio, Shanghai, China) [19]. Briefly, after transfection with WISP2 plasmid or siRNAs in ESCC cells in different groups, the cells were harvested, washed with PBS and resuspended in the binding buffer provided by the kit. The cells were then labeled with annexinV- FITC/ PI and the percentage of apoptosis cells was quantified using the Multicycle AV software (FACSAria, BD Biosciences, CA, USA).

### Western blotting

Western blotting was performed according to the established methods [20, 21]. The transfected ESCC cells were rinsed twice with ice-cold phosphate buffered saline (PBS, pH 7.4), lysed in a cooled buffer, and sonicated for 5 s on ice. Then the lysates were boiled in a water bath for 10 min, and centrifuged at 12,000 g for 10 min at 4 °C. The protein concentrations in the supernatant were measured by BCA kit (Pierce, USA). Equal amounts of proteins were separated by 10% SDS-polyacrylamide gel electrophoresis (SDS-PAGE) and transferred to nitrocellulose membranes (Amersham, Germany).The protein expression was measured as described previously [20, 21]. .

### Transwell invasion assay

The migration and invasion assays of transfected ESCC cells were performed using Transwell cell-culture chambers (Corning, USA). For invasion assay, the chamber was coated with the admixture of Matrigel (BD, USA). The treated cells were seeded into the upper chamber with serum-free medium (5 × 10^4^ cells), and the bottom chamber contained DMEM medium with 10% FBS. When the cells invaded for 20 h, the cells were washed, fixed, stained with Calcein-AM for 20 min or crystal violet. Then the invaded cells were counted and taken pictures under microscope (Hanrong Company, Shanghai).

### In vivo experiments

The animal experiments were operated following the previous description [22]. To establish all the nude mice models of ESCC which were divided into three groups, Eca109 control group, Eca109-empty vector group, Eca109-WISP2 expression. We also injected Eca109 cells with control siRNA or WISP2 siRNA transfection to nude mice. To choose the right axillary injection amount of cell suspension, regular measurement of tumor growth in volume, record and process data, and draw the tumor growth curve. After 28 days, the nude mice were sacrificed by cervical dislocation, and tumor tissues were taken out. Tumor weights were measured. The expression of WISP2, E-cadherin, ERK1/2, and Slug was measured by Western blotting analysis.

### Statistical analysis

All data were expressed as mean ± SD. Comparisons between groups were performed using one-way ANOVA followed by Tukey’s post-hoc test. Differences were evaluated using Student’s t test. Statistical significance was indicated as *p*-value < 0.05. Statistical tests were performed with GraphPad Prism version 5 for Windows (GraphPad Software, San Diego, California, USA).

## Results

### Expression of WISP2 is associated with clinical significance in ESCC

**Fig. 1:**
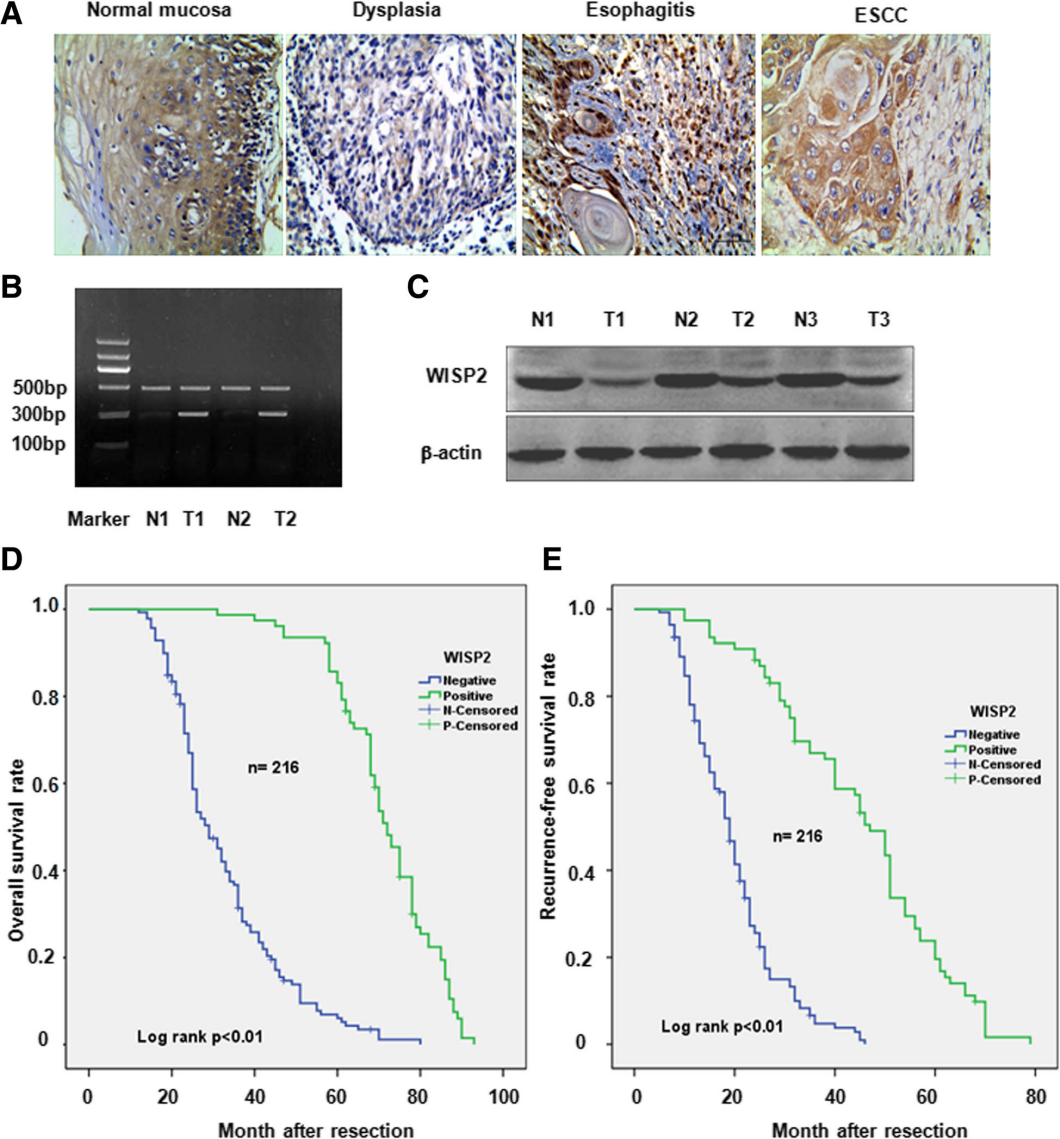
Immunohistochemical staining of WISP2 protein in ESCC tissues. **a**, Immunohistochemical staining images of WISP2 in esophageal normal mucosa (left panel), low-differentiated squamous cell carcinoma tissue and esophagitis tissue (middle panel), and high-differentiated squamous cell carcinoma tissues (right panel). **b**, RT-PCR was used to measure the WISP2 mRNA level in ESCC tissues and non-tumor tissues. N1: normal mucosa tissue 1; N2: normal mucosa tissue 2; T1: ESCC tissue 1; T2: ESCC tissue 2. **c**, Western blotting was used to measure the WISP2 protein level in ESCC tissues and non-tumor tissues. N1–3: normal mucosa tissue 1–3; T1-T3: ESCC tissue 1–3. **d-e**, The survival curves for WISP2 in ESCC patients with overall survival rate (**d**) and recurrence-free survival rate (**e**)

To explore the association of WISP2 and clinicopathologic significance in ESCC, we measured the protein of WISP2 in 216 cases of ESCC and 60 cases of normal esophageal epithelium by immunohistochemistry method (Table 1). We found that the positive expression rate of WISP2 protein was 35.65% (77/216) in 216 cases of ESCC tissues. 64.35% tumor tissues have negative expression of WISP2, suggesting that lower expression of WISP2 was exhibited in ESCC tissues. 51.66% of positive expression of WISP2 in 60 cases of esophageal normal mucosa was observed (Table 2). Compared with the positive expression rate of WISP2 in esophageal normal mucosa tissues (Fig. 1A left panel) esophageal dysplasia tissues (Fig. 1A middle left panel), and esophagitis tissue (Fig. 1A middle right panel), WISP2 level in ESCC tissues was downregulated (Fig. 1A right panel). The expression of WISP2 was associated with tumor lymph node metastasis, depth of tumor infiltration, histological differentiation and TNM stage (Table 1), but was not associated with the patient’s gender, age, tumor location, the general type, and diameter (Table 1). To further validate our result, the mRNA of WISP2 was detected by Reverse Transcription PCR (RT-PCR) in 28 cases of ESCC and adjacent normal tissues. We found that there was a significant difference for WISP2 mRNA level between adjacent normal tissues and ESCC tumors (Fig. 1B and Table 3). Our western blotting results showed down-regulation of WISP2 in ESCC tumor tissues compared with adjacent normal tissues (Fig. 1C). Furthermore, we investigated the relationship between WISP2 and survival in 93 cases of ESCC. Our Kaplan-Meier curves analysis (log-rank test) showed that there was a significance difference between the positive expression of WISP2 groups and the negative expression of WISP2 groups in overall survival rate (Fig. 1D and Table 4) and recurrence-free survival rate (Fig. 1E). Taken together, lower expression of WISP2 in ESCC tissues was associated with lymph node metastasis, depth of invasion and the stage.

**Table 3:** Expression of WISP2 mRNA in normal esophageal mucosa and ESCC

Parameter	Cases	$\overline{x}$± s	t	*P*
Normal mucosa	28	0.830 ± 0.027	−17.161 < 0.01	
Tumor tissue	28	0.452 ± 0.114

**Table 4:** The statistics and univariate analysis of the patients with ESCC

Parameter	Cases	5-y survival rate (%)	χ^2^	*P*
WISP2			146.25	< 0.01
+	77	85.7		
–	139	5.76		

### Overexpression of WISP2 inhibits cell growth and induces apoptosis

**Fig. 2:**
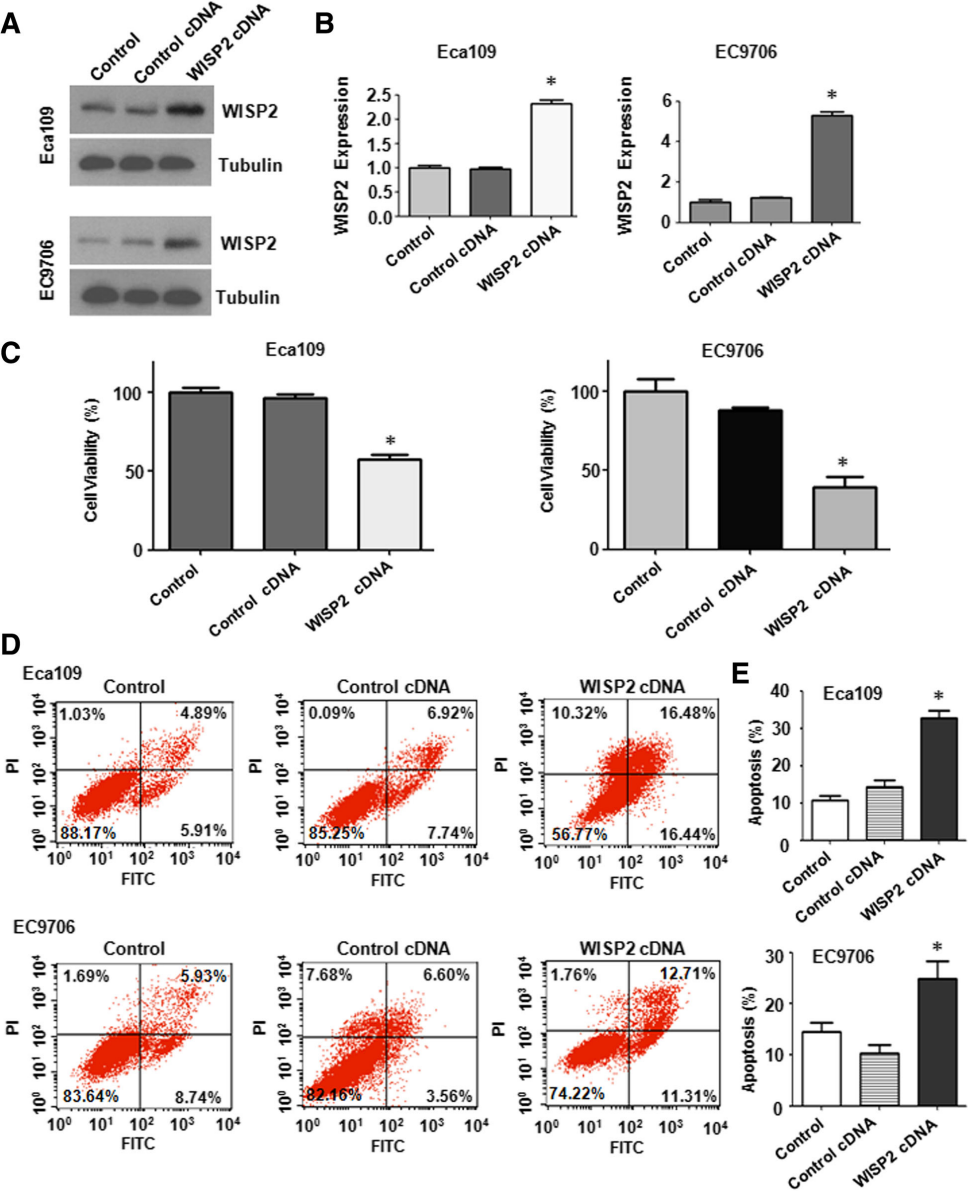
Over-expression of WISP2 inhibits cell proliferation and induces apoptosis. **a**, Western blot analysis was used to measure the WISP2 expression in ESCC cells transfected with WISP2 cDNA.**b**, Quantitative results for the panel A. * *P* < 0.01, vs Control. **c**, MTT assay was used to measure cell proliferation in ESCC cells after WISP2 cDNA transfection. * *P* < 0.05 vs Control. **d**, Flow cytometry was used to measure cell apoptosis in ESCC cells after WISP2 cDNA transfection. E, Quantitative results for cell apoptosis percentage in ESCC cells after WISP2 cDNA transfection.. * *P* < 0.05, vs Control

In order to explore the role of WISP2 in ESCC, the plasmid with WISP2 cDNA was transfected into ESCC cells. The efficacy of WISP2 cDNA transfection for overexpression of WISP2 in ESCC cells was validated by Western blotting analysis. Our result showed that WISP2 was significantly overexpressed in ESCC cells after cDNA transfection (Fig. 2A and B). MTT assay was used to measure cell growth in WISP2-overexpressing ESCC cells. We found that overexpression of WISP2 led to 45% cell growth inhibition in Eca109 cells (*p* = 0.007) and 55% growth inhibition in EC9706 cell (*p* = 0.002) compared with control cDNA transfection group (Fig. 2C). To further characrized the function of WISP2 in ESCC cells, we measured the cell apoptotic death by Annexin V-FITC/PI method in ESCC cells after WISP2 overexpression. We found that upregulation of WISP2 increased the percentages of apoptotic cells from 14.56% in control cDNA transfection group to 32.92% in Eca109 cells with WISP2 cDNA transfection (*p* = 0.002), and from 10.16% in control cDNA group to 24.02% in EC9706 (*p* = 0.012) cell line (Fig. 2D and E). This data implied that WISP2 suppressed cell growth partly due to induction of apoptosis in ESCC cells.

### Overexpression of WISP2 retards cell migration and invasion

**Fig. 3:**
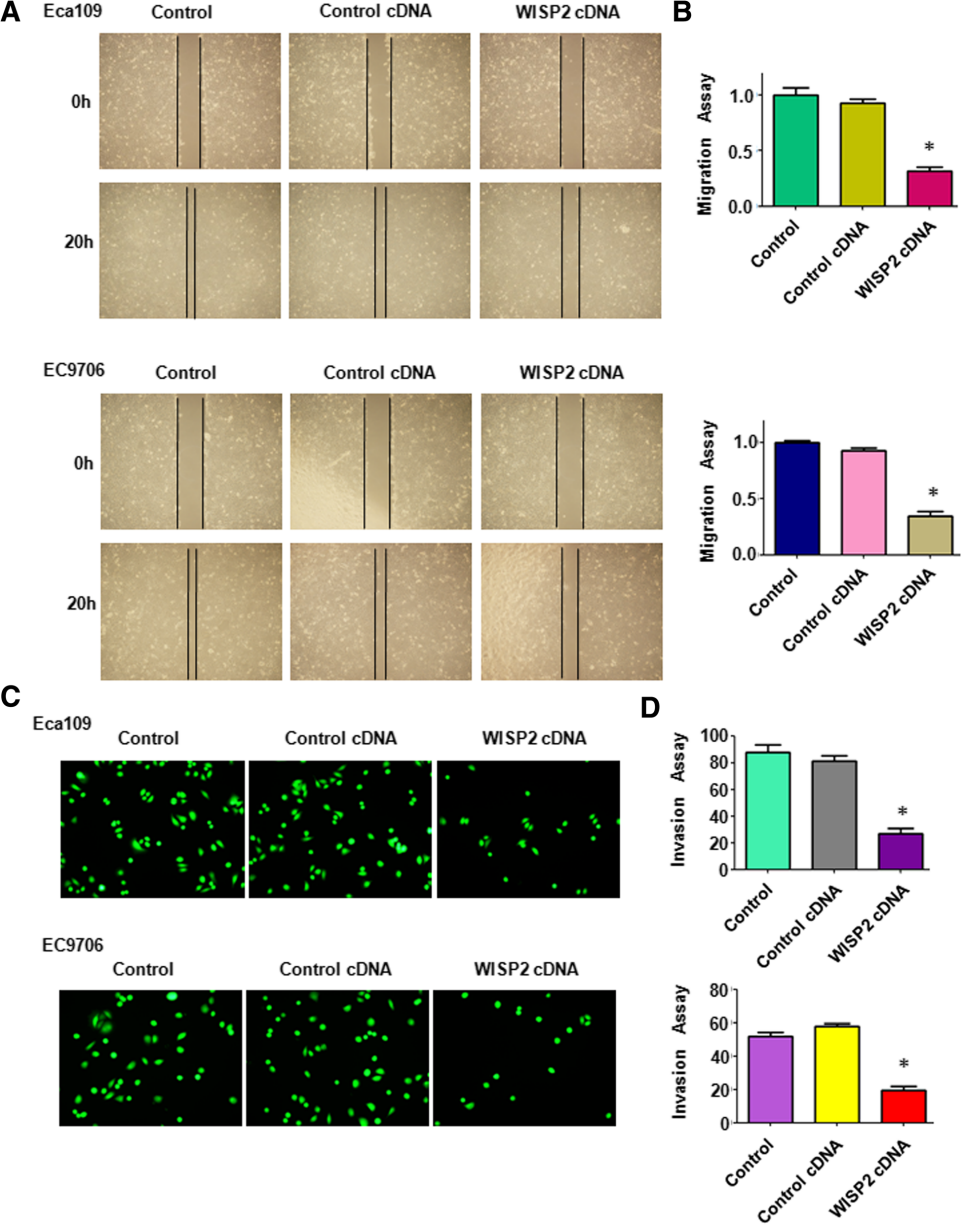
Over-expression of WISP2 inhibits cell migration and invasion. **a**, Wound healing assays was used to detect the cell migration in ESCC cells after WISP2 cDNA construct transfection. **b**, Quantitative results for the panel A. * *P* < 0.05, vs Control. **c**, Invasion assays were used to measure the cell invasion in ESCC cells following WISP2 cDNA construct transfection. **d**, Quantitative results for the panel C. * *P* < 0.05, vs Control

**Fig. 4:**
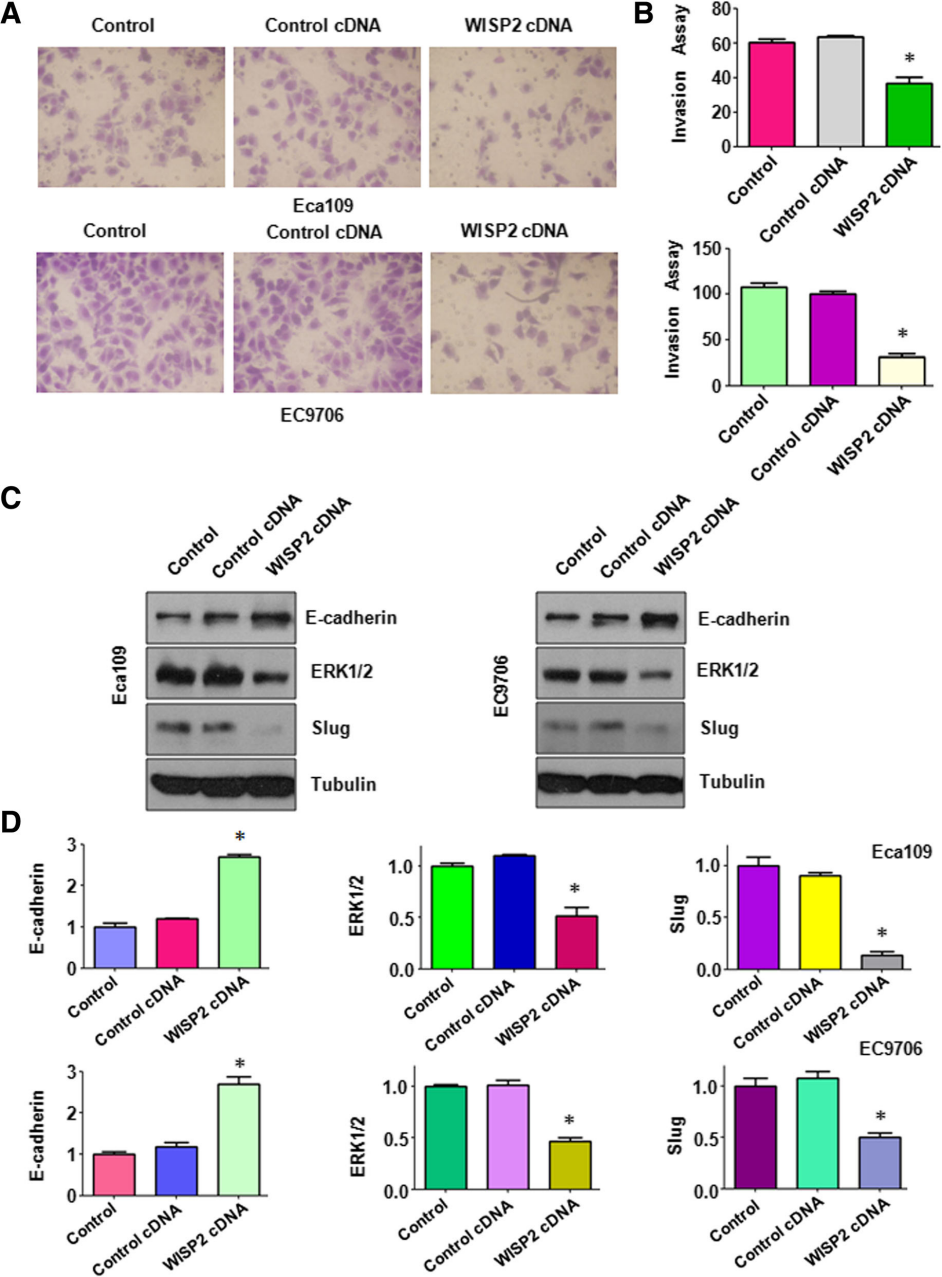
Over-expression of WISP2 regulates E-cadherin, ERK1/2, and Slug. **a**, Invasion assays were used to measure the cell invasion in ESCC cells following WISP2 cDNA construct transfection. **b,** Quantitative results for the panel A. * *P* < 0.05, vs Control. **c**, Western blot analysis was used to measure the expression of E-cadherin, ERK1/2, and Slug in ESCC cells transfected with WISP2 cDNA. **d**, Quantitative results for the panel C. * *P* < 0.05, vs Control

Next, we examined whether WISP2 could regulate cell migration and invasion in ESCC cells. Wound healing assay was performed to detect the migration of ESCC cells after WISP2 overexpression. We found that up-regulation of WISP2 inhibited 60 to 70% cell migration in Eca109 cells (*p* = 0.009) and EC9706 (*p* = 0.002) cell lines (Fig. 3A and B). Our matrigel invasion assay results showed that overexpression of WISP2 remarkably retarded 65 to 70% cell invasion in Eca109 cells (*p* = 0.002) and EC9706 (*p* = 0.007) cell lines (Fig. 3C-3D). Similarly, the invaded cells with WISP2 overexpression that stained with crystal violet also were reduced to 50% in Eca109 cells (*p* = 0.0035) and 30% cell invasion in EC9706 (*p* = 0.0016) cell lines, respectively (Fig. 4A-4B). Our findings indicate that WISP2 overexpression retarded cell migration and invasion in ESCC cells.

### Overexpression of WISP2 decreases ERK expression and increases E-cadherin level

To further determine the mechanism of WISP2-mediated tumor suppression, we investigated whether overexpression of WISP2 could regulate ERK, Slug, and E-cadherin in ESCC cells. Our Western blotting data showed that E-cadherin level was dramatically upregulated in the WISP2 cDNA transfected cells (Fig. 4C and D). We also observed that the expression of ERK and Slug was downregulated in cells after WISP2 cDNA transfection in ESCC cells (Fig. 4C and D). These data indicated that overexpression of WISP2 exerted its tumor suppressive function partly via inhibition of ERKand Slug, but upregulation of E-cadherin in ESCC cells.

### Downregulation of WISP2 enhances cell growth and inhibits apoptosis

**Fig. 5:**
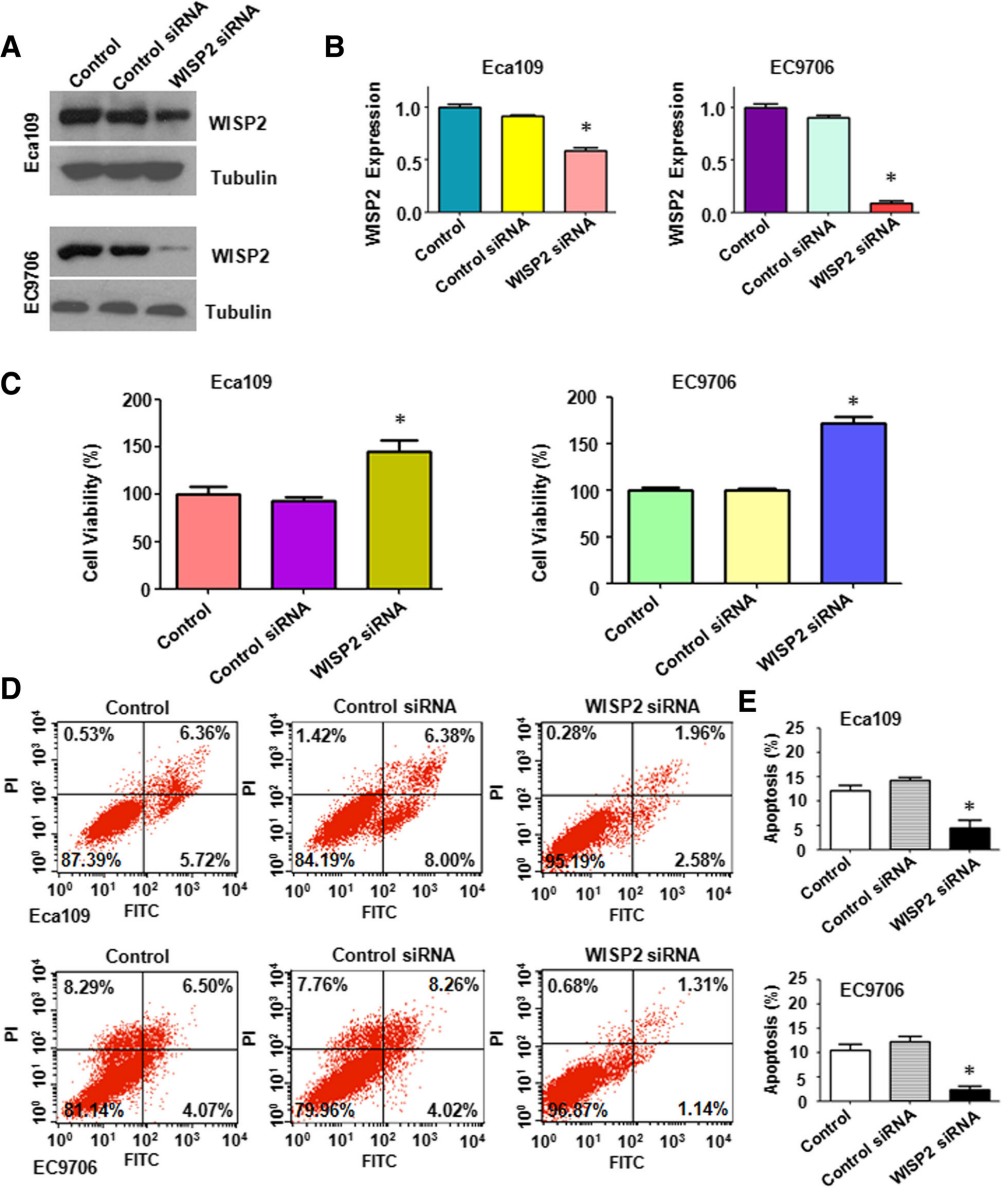
Down-regulation of WISP2 enhances cell proliferation and inhibits apoptosis. **a**, Western blot analysis was used to measure the WISP2 expression in ESCC cells transfected with WISP2 siRNA. **b**, Quantitative results for the panel A. * *P* < 0.01, vs Control. **c**, MTT assay was used to measure cell proliferation in ESCC cells following WISP2 siRNA transfection. * *P* < 0.05 vs Control. **d**, Flow cytometry was used to measure cell apoptosis in ESCC cells following WISP2 siRNA transfection. * *P* < 0.05 vs Control. E, Quantitative results for cell apoptosis percentage in ESCC cells after WISP2 siRNA transfection.. * *P* < 0.05, vs Control

To further validate the function of WISP2, ESCC cells were transfected with WISP2 siRNA or control siRNA. The efficacy of WISP2 siRNA transfection was measured by Western blotting. Our result showed that WISP2 siRNA transfection significantly reduced the expression of WISP2 in both ESCC cell lines (Fig. 5A and B). Cell growth was determined by MTT in ESCC cells after WISP2 siRNA transfection. We found that down-regulation of WISP2 promoted 50% cell growth in Eca109 cells (*p* = 0.0027) and 70% cell growth in EC9706 cell (*p* = 0.0004) compared with control siRNA transfection group (Fig. 5C). In line with this, we found that down-regulation of WISP2 decreased the percentages of apoptotic cells from 14.38% in control siRNA transfection group to 4.54% in Eca109 cells with WISP2 siRNA transfection (*p* = 0.0028), and from 12.28% in control siRNA group to 2.45% in EC9706 (*p* = 0.0012) cell line (Fig. 5D). These findings indicate the tumor suppressive role of WISP2 in ESCC cells.

### Downregulation of WISP2 promotes cell migration and invasion

**Fig. 6:**
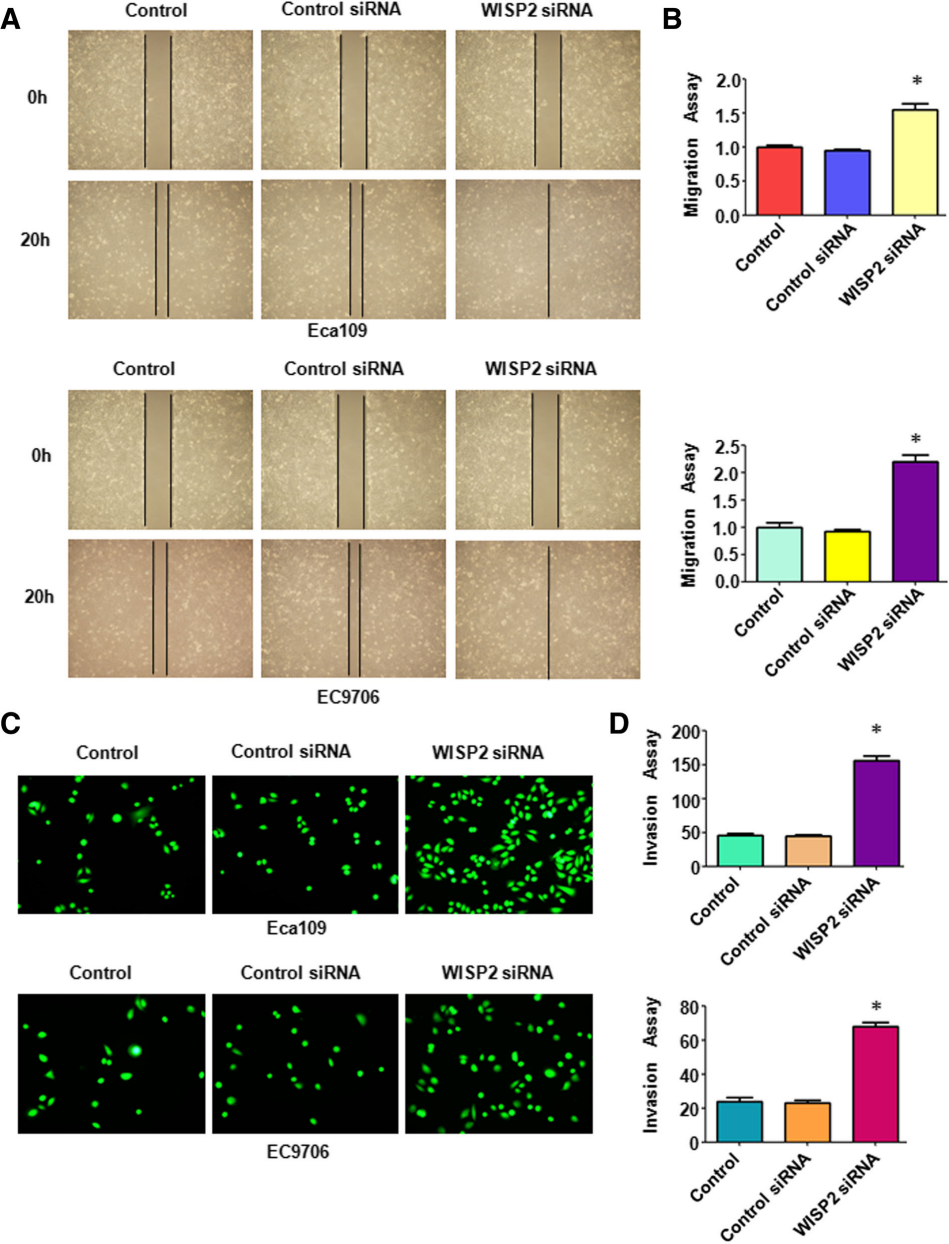
Down-regulation of WISP2 increase cell migration and invasion. **a**, Wound healing assays was used to detect the cell migration in ESCC cells after WISP2 siRNA transfection. **b**, Quantitative results for the panel A. * *P* < 0.05, vs Control. **c**, Invasion assays were used to measure the cell invasion in ESCC cells following WISP2 siRNA transfection. **d**, Quantitative results for the panel C. * *P* < 0.05, vs Control

**Fig. 7:**
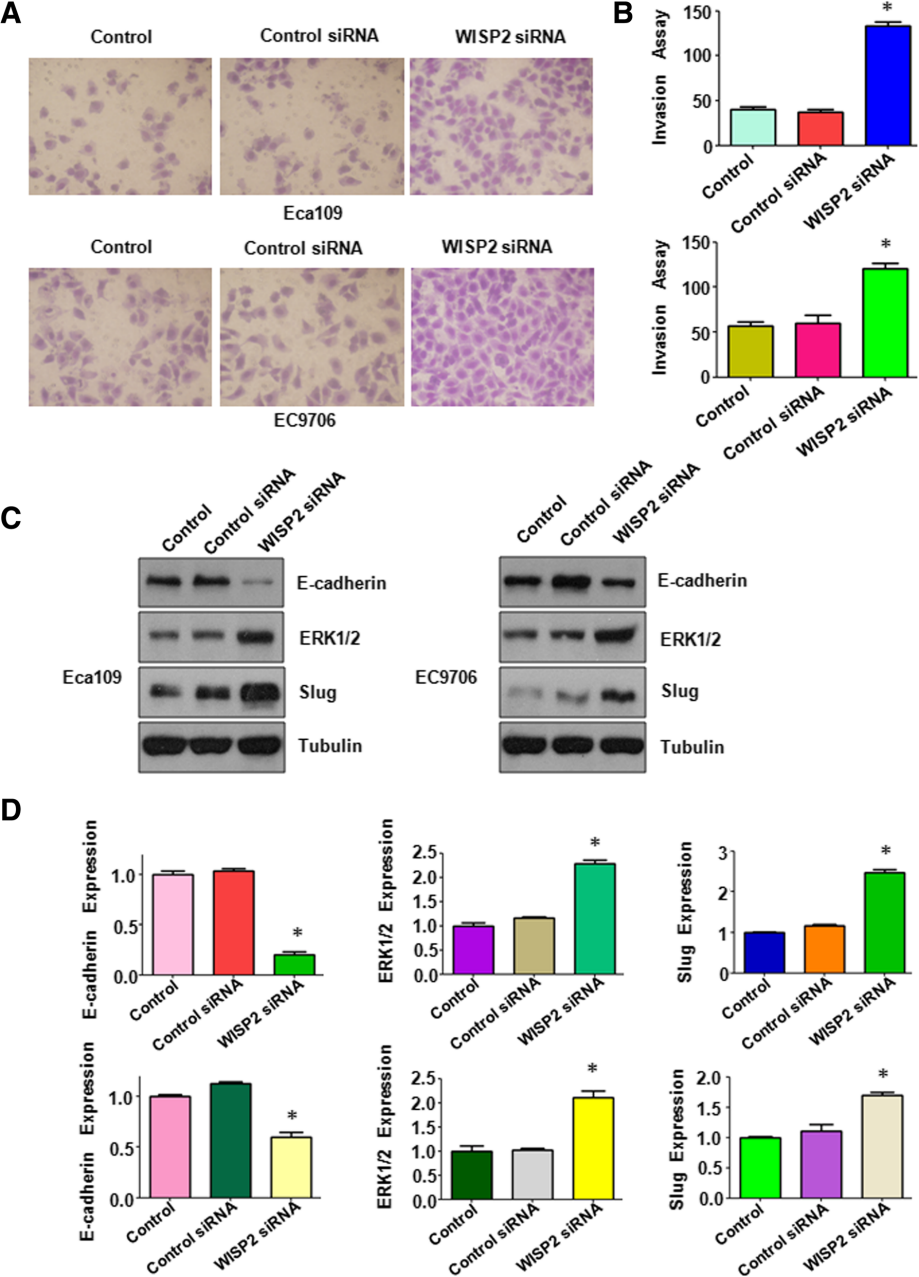
Down-regulation of WISP2 regulates E-cadherin, ERK1/2, and Slug. **a**, Invasion assays were used to measure the cell invasion in ESCC cells following WISP2 siRNA transfection. **b**, Quantitative results for the panel A. * *P* < 0.05, vs Control. **c**, Western blot analysis was used to measure the expression of E-cadherin, ERK1/2, and Slug in ESCC cells transfected with WISP2 siRNA. **d**, Quantitative results for the panel C. * *P* < 0.05, vs Control

To further dissect the role of WISP2 in cell migration and invasion, wound healing assay was applied for detecting cell migration in cells after downregulation of WISP2 in ESCC cells. Our wound healing assay results showed that WISP2 siRNA transfection led to an increase of cell wound healing in Eca109 cells (*p* = 0.0007) and EC9706 (*p* = 0.0003) cells (Fig. 6A and B). Moreover, Transwell chamber assay was conducted to measure the cell invasion in ESCC cells after WISP2 siRNA transfection. We found that WISP2 siRNA transfected cells showed 3–3.5 folds promotion of cell invasion in both Eca109 cells (*p* < 0.001) and EC9706 (*p* = 0.0011) compared to control siRNA transfected cells (Fig. 6C-D). In line with this, WISP2 downregulation led to a significant increase in cell invasion of Eca109 cells (*p* = 0.0288) and EC9706 (p < 0.001) cells (Fig. 7A-B). Mechanistically, downregulation of WISP2 decreased E-cadherin level and increased the level of ERK1/2 and Slug in ESCC cells (Fig. 7C-D).

### Overexpression of WISP2 inhibits tumor growth in vivo

**Fig. 8:**
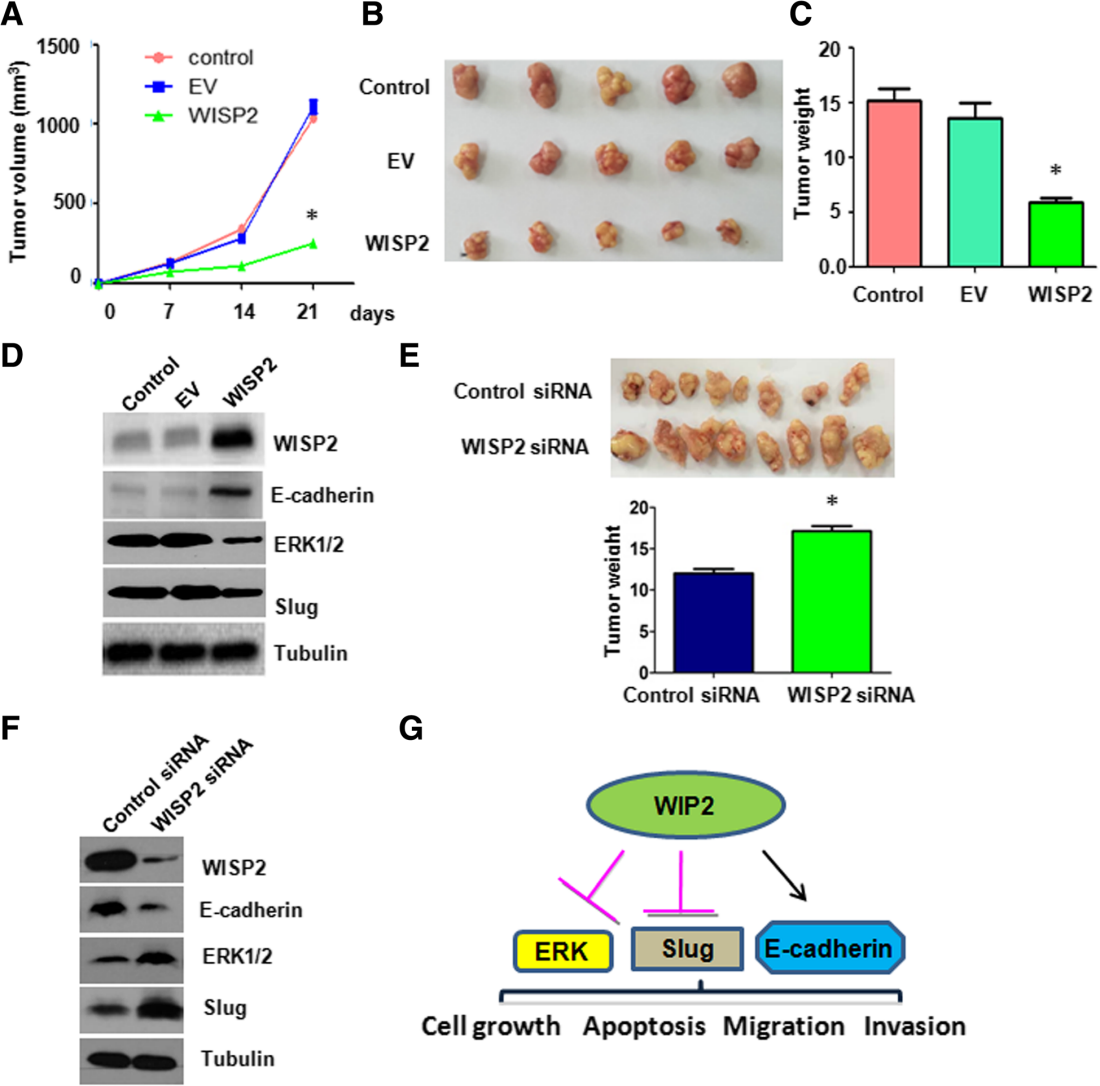
Overexpression of WISP2 inhibits tumor growth in vivo. **a**, Tumor volumes were measured in nude mice after injection with Eca109 cells with WISP2 overexpression. **b**, Tumor sizes in nude mice after injection with Eca109 cells with WISP2 overexpression were photographed. **c**, Tumor weights were measured in nude mice after injection with Eca109 cells with WISP2 overexpression. **d**, Western blot analysis was used to measure the expression of WISP2, E-cadherin, ERK1/2, and Slug in tumor tissues. **e**, Tumor sizes in nude mice after injection with Eca109 cells with WISP2 downregulation were photographed (Top panel). Tumor weights were measured in these mouse tissues (Bottom panel).**f**, Western blot analysis was used to measure the expression of WISP2, E-cadherin, ERK1/2, and Slug in tumor tissues.**g**, A flowchart to show how WISP2 regulates the expression of E-cadherin, ERK1/2, and Slug in ESCC

The in vivo results showed that overexpression of WISP2 inhibited tumor growth using nude mouse model of ESCC (Fig. 8A). Tumor growth volume in WISP2 overexpression group was lower than control group and empty vector group (Fig. 8B). Consistently, the final tumor weight of WISP2 overexpressing stable cell group was lower than that of control groups (Fig. 8C). Furthermore, the expression of WISP2 and E-cadherin at protein level was increased in WISP2 cDNA transfection group, while the expression of ERK1/2 and Slug was decreased in WISP2 overexpressing group (Fig. 8D). Consistently, down-regulation of WISP2 retarded tumor growth in mice (Fig. 8E). Downregulation of WISP2 led to a decrease in E-cadherin expression and an increase in ERK1/2 and Slug level in mice tissues (Fig. 8F). Taken together, WISP2 could be a tumor suppressor via regulation of E-caherin, Slug, and ERK1/2 in ESCC (Fig. 8G).

## Discussion

To date, the role of WISP2 in ESCC is unelucidated, although the function of WISP2 was investigated in a variety of human cancers [14, 23, 24]. In the present study, we reported that WISP2 overexpression inhibited cell growth and induced cell apoptosis, suppressed cell migration and invasion in ESCC cells. Moreover, WISP overexpression retarded tumor growth in mouse model. WISP2 downregulation enhanced cell growth, inhibited apoptosis, promoted cell migration and invasion in ESCC cells. Mechanistically, WISP2 exerts its tumor suppressive functions via regulation of ERK1/2, Slug, and E-cadherin in ESCC cells. Our study provides the new evidence showing WISP2 as a tumor suppressor in ESCC.

WISP2 has been reported to govern several gene expressions in human cancer cells. For instance, downregulation of WISP2 promoted PD-L1 level in breast cancer cells. Inactivation of PD-L1 restored the susceptibility of resistant cells with WISP2 downregulation to CTL treatment [25]. One study reported that WISP2 inhibited PI3K/Akt pathway and Skp2 expression, and subsequently increased p27 accumulation, induced FOXO3a expression and activity [11]. Further, loss of WISP2 enhanced cancer stem-like phenotype characterized and increased the level of stem cell markers Nanog and Oct3/4, and activated TGF-β pathway in breast tumor cells [26]. WISP2 recombinant protein triggered MET (mesenchymal-epithelial transition) in pancreatic cancer cells [15]. HBx (hepatitis B virus X gene) mutants, frequently happened in HBV (hepatitis B virus)-related hepatocellular carcinoma, enhanced cell proliferation and migration via regulation of Wnt/β-catenin signaling pathway [27]. HBx mutants stabilized β-catenin level via inhibition of GSK3β in HCC cells, resulted in increased WISP2 and c-Myc [27]. The expression level of WISP2 was enhanced in overexpressing TCF-4J (T-cell factor-4 isoform J) HCC cells, indicating that WISP2 could play a potential role in HCC progression [28].

WISP2 downregulation promoted cell growth, migration and invasion, but WISP2 overexpression suppressed cell metastasis through regulation of EMT and inhibition of MMP-9 and MMP-2 via ERK in gastric cancer cells [13]. It has been reported that WISP2 could regulate the Wnt/β-catenin signaling pathway in gastric cancer [14]. In line with these reports, our study dissected that WISP2 overexpression decreased expression of ERK1/2. WISP2 could inhibit the proliferation and induced apoptosis by suppressing ERK1/2 signal in ESCC. E-cadherin is a key molecule to be involved in EMT and cell invasion [29, 30]. WISP2 was reported to control migration and invasion via regulation of Snail and E-cadherin in breast cancer cells [10]. Our study also showed that WISP2 overexpression increased E-cadherin level and decreased Slug level in ESCC. Thus, WISP2 inhibit the invasion and migration by down-regulating slug and up-regulating E-cadherin expression in ESCC.

## Conclusions

WISP2 plays a tumor suppressor role in ESCC cells. WISP2 overexpression inhibited cell growth and induced cell apoptosis, slowed cell migration and invasion in ESCC cells whereas downregulation of WISP2 has opposed effects. Mechanistically, WISP2 exerts its anti-tumor functions via regulation of ERK1/2, Slug, and E-cadherin in ESCC cells. Notably, WISP2 overexpression is associated with survival in ESCC patients. Our findings suggest that activation of WISP2 could be a potential therapeutic strategy for the treatment of ESCC. Without a doubt, further investigations are required to define the role of WISP2 using conditional knockout or knockin transgenic mouse model. The detailed molecular mechanisms how WISP2 is regulated and its downstream targets in ESCC are needed to be explored in the future.

## Abbreviations

ERK: Extracellular signal-regulated kinase
ESCC: Esophageal squamous cell carcinoma
HBV: Hepatitis B virus
HBx: Hepatitis B virus X gene
MET: Mesenchymal-epithelial transition
MMP-2: Matrix metalloproteinase-2
PI3K: Phosphatidylinositol 3-kinase
Skp2: S-phase kinase associated protein 2
TCF-4J: T-cell factor-4 isoform J
WISP2: WNT1 inducible signaling pathway protein 2
